# The Relevance of Preoperative Identification of the Adamkiewicz Artery in Posterior Mediastinal Pediatric Tumors

**DOI:** 10.1245/s10434-021-10381-8

**Published:** 2021-07-31

**Authors:** Andreas Schmidt, Johann-Martin Hempel, Verena Ellerkamp, Steven W. Warmann, Ulrike Ernemann, Jörg Fuchs

**Affiliations:** 1 Department of Pediatric Surgery and Pediatric Urology, University Children’s Hospital, Eberhard Karls University Tuebingen, Tübingen, Germany; 2 Department of Diagnostic and Interventional Neuroradiology, University Hospital, Eberhard Karls University Tuebingen, Tübingen, Germany

## Abstract

**Background:**

Injury to the artery of Adamkiewicz (AKA) during surgery may lead to spinal cord ischemia and severe neurologic complications. Posterior mediastinal tumors may be adjacent to AKA, but data on preoperative visualization of AKA in children are rare. This study analyzed the importance of identifying the AKA preoperatively by spinal digital subtraction angiography (DSA) in children with posterior mediastinal tumors for therapeutic procedure.

**Methods:**

Between 2002 and 2021, 36 children with posterior mediastinal tumors were evaluated for surgery at the authors’ clinic. In 10 children with left-sided or bilateral tumor located at vertebral levels T8 to L1, spinal DSA was performed during preoperative workup to assess AKA. The patient and tumor characteristics as well as the diagnostic and therapeutic procedures were analyzed.

**Results:**

The median age of the 10 children at examination was 69 months (range, 16–217 months). Three of the children were younger than 2 years. The tumor entities were neuroblastoma, ganglioneuroblastoma, ganglioneuroma, local relapse of a hepatocellular carcinoma, and neurofibroma. The AKA was identified in all cases, and proximity to the tumor was detected in four patients, three of whom had their planned surgery changed to irradiation. No complications occurred during spinal DSA or surgery.

**Conclusions:**

In posterior mediastinal pediatric tumors, spinal DSA is a safe and reliable method for preoperative visualization of the AKA. It can show proximity to the tumor and guide the local therapy, thereby avoiding critical intra- and postoperative situations.

Tumors in the posterior mediastinum in children account for approximately one third of all mediastinal tumors.^1^ They comprise a variety of types,^2^,^3^ but approximately 90% are of neurogenic origin, with neuroblastoma as the most common tumor.^4^,^5^

Neurogenic tumors originate from the sympathetic nervous system and peripheral nerves. Therefore, they often are located paravertebrally in the vicinity of the vascular system that ensures blood supply to the spinal cord. Injury to the vasculature during surgery may result in decreased spinal cord perfusion and ischemia, leading to paraplegia and paraparesis.^6^

Of particular importance is the artery of Adamkiewicz (AKA).^7^,^8^ The AKA is located between vertebral levels T3 and L5, but in 89% of patients, it is located between levels T8 and L1, and 76.6% of AKAs arise from the left side.^9^

Specific reference is made to the AKA in the International Neuroblastoma Risk Group Staging System (INRGSS).^10^ Because of possible injury to the AKA during surgery, infiltration of the costovertebral junction between vertebral levels T9 and T12 is defined as an image-defined risk factor (IDRF).^11^ However, reports on surgical complications in cooperative neuroblastoma studies and metanalyses provide only overall incidences without giving more specific details for posterior mediastinal tumors, particularly concerning injury to the AKA.^12^–^19^

Ultimately, data on impairment of neurologic function due to spinal cord ischemia after surgery for posterior mediastinal tumors in children are limited to case reports.^20^–^23^ Even less information is available on the demonstration of AKA to guide the surgical procedure. After experiencing a postoperative neurologic deficit in a child after surgery of posterior mediastinal tumors, Boglino et al.^20^ and Nordin et al.^23^ recommended that spinal diagnostic subtraction angiography (DSA) be performed when the AKA is at risk, but only Nordin et al.^23^ described spinal DSA performed in two children.

This study presents the largest case series of preoperative spinal DSA in children with solid tumors in the posterior mediastinum to date. We discuss the criteria for applying the method and describe the impact of its implementation on an unselected patient cohort.

## Methods

### Study Design and Patients

The patient records of all the children evaluated for surgery of posterior mediastinal tumors at our clinic from 2002 to 2021 were screened. The parameters analyzed were patient age, tumor type and location, spinal DSA, surgery, diagnostics and surgery complications, and outcome. The patient data are presented as median and range. The study was approved by the Ethical Committee of the Medical Faculty of the University of Tuebingen and the University Hospital Tuebingen, Germany (ref. 947/2020BO2).

### Selective Spinal DSA

All the children had already received magnetic resonance imaging (MRI) or computed tomography (CT) scan before spinal DSA for diagnosis and planning for surgery. Spinal DSA was performed if the tumor extended to vertebral levels T8 to L1 on the left side. For spinal DSA, we used the biplane Siemens Artis zee with the as40HDR HDR flat detector system, the MEGALIC Cat Plus x-ray tube, and Automatic Exposure Control (AED; Siemens Healthineers, Erlangen, Germany). Spinal angiography was performed with the patient under general anesthesia using a pediatric femoral sheath and 4-French diagnostic catheters.

## Results

### Patient Characteristics

A total of 36 patients with posterior mediastinal tumors were evaluated for surgery. All the patients were of caucasian ethnicity. In 10 patients (4 girls and 6 boys), the left-sided or bilateral tumor extended into vertebral levels T8 to L1. Spinal DSA was performed for these patients. The tumors were neuroblastoma (*n* = 4), ganglioneuroblastoma (*n* = 2), ganglioneuroma (*n* = 2), local relapse of a hepatocellular carcinoma (*n* = 1), and neurofibroma (*n* = 1). The median age at the time of spinal DSA was 69 months (range, 16–217 months), and three of the children were younger than 2 years.

### Tumor and AKA Location

The tumors were located between vertebral levels T3 and L2 (Table 1) on the left side or bilaterally. The AKA was detected in all cases. It originated at T7 to L2/3, most often at L1 (*n* = 3). Seven AKAs were located on the left side and three on the right side. Tumor and AKA were at the same level and side in four patients (patients 4, 6, 7, and 9) and at a different level or side in six patients (patients 1, 2, 3, 5, 8, and 10) (Table 1).

**Table 1:** Characteristics of tumor and artery of Adamkiewicz (AKA)

Patient	Diagnosis	Location of tumor		Location of AKA		Age at angiography (months)
		Level	Site	Level	Site	
1	Ganglioneuroblastoma	T5–L2	Bilateral	L2/3	Right	23
2	Ganglioneuroblastoma	T8–T10	Left	T12	Left	16
3	Neuroblastoma	T3–T11	Left	L1	Left	19
4	Local relapse of HCC	T12–L2	Left	L1	Left	217
5	Ganglioneuroma	T6–T8	Left	L1	Left	60
6	Neuroblastoma	T7–10	Left	T9	Left	79
7	Ganglioneuroma	T8–T12	Left	T11	Right	133
8	Neurofibroma	T7–T11	Left	T10	Left	188
9	Neuroblastoma	T4–T12	Left	T7	Left	78
10	Neuroblastoma	T5–L1	Left	T9	Right	56

*HCC* hepatocellular carcinoma

### Therapy and Outcome

**Fig. 1:**
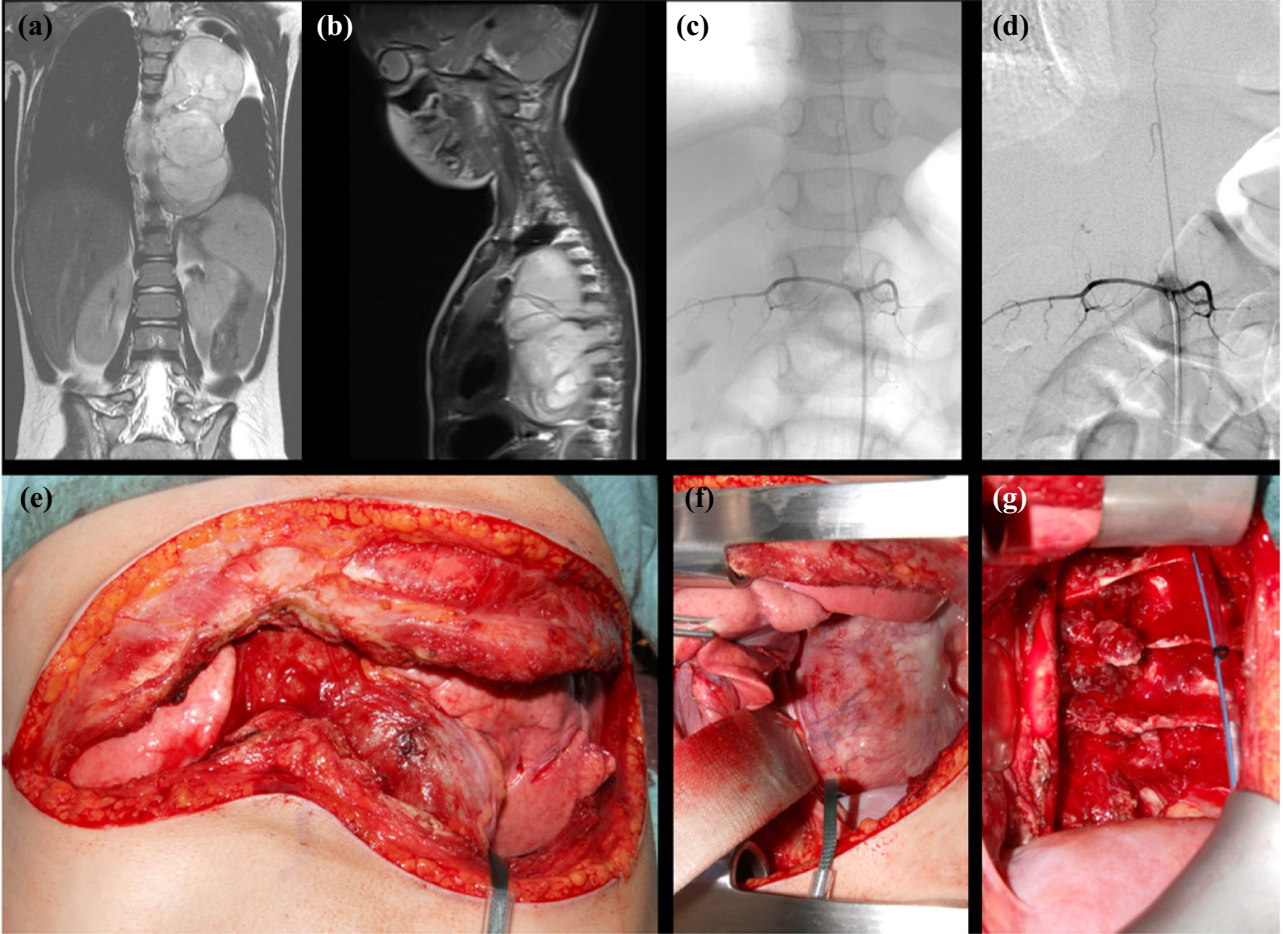
Left-sided, thoracic ganglioneuroma in a 5-year-old girl (patient 5). (**a**) Coronar and (**b**) sagittal magnetic resonance (MR) images. The tumor is located from T6 to T8. (**c**) Coronar and (**d**) sagittal images of selective spinal angiography. The origin of the artery of Adamkiewicz at level L1 is on the left. (**e**) Clamshell incision for tumor resection. (**f**) Paravertebrally located tumor. (**g**) Situs after tumor resection.

Surgery was performed as planned for five patients with differing locations of AKA and tumor (shown for patient 5; Fig. 1). The tumor of one patient (patient 3) showed spontaneous regression, so that in accordance with the treatment protocol, local therapy was not applied. In the four children with coincident location of the origin of the AKA and tumor, the individual risk assessment led to different therapeutic strategies.

**Fig. 2:**
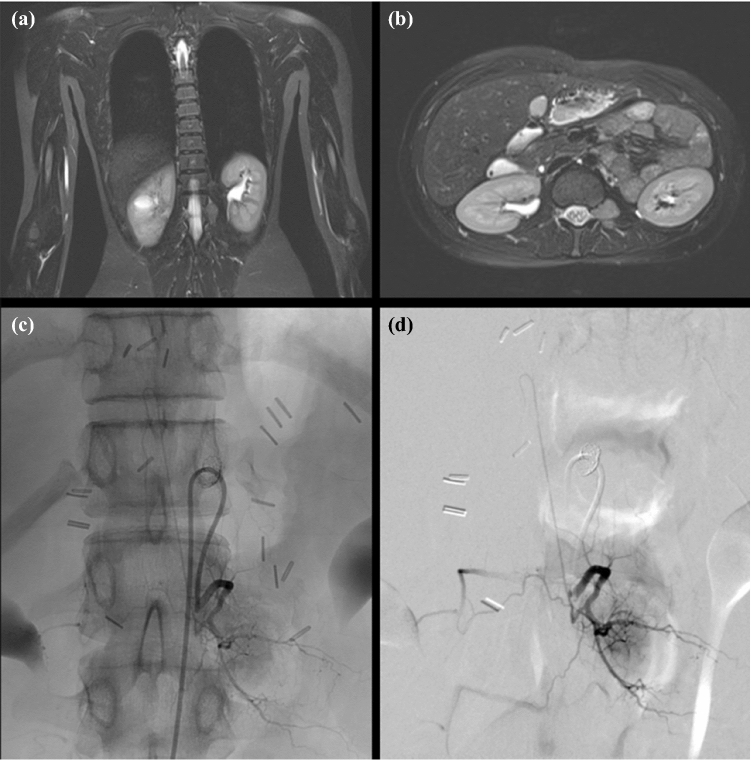
Left-sided, paravertebral local relapse of hepatocellular carcinoma in an 18-year-old girl (patient 4). (**a**) Coronar and (**b**) axial MR images. The tumor is located from T12 to L2. (**c**) Coronar and (**d**) sagittal images of selective spinal angiography. The origin of the artery of Adamkiewicz is at level L1 on the left. Surgery was abandoned, and proton beam therapy was performed.

**Fig. 3:**
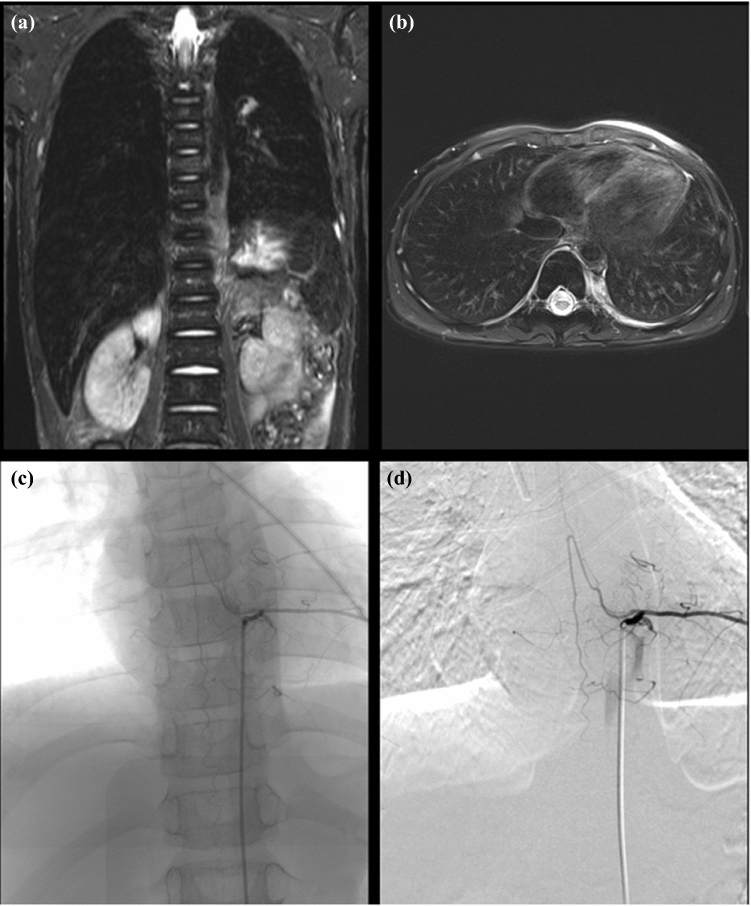
Left-sided, thoracic neuroblastoma in a 6-year-old boy (patient 6). (**a**) Coronar and (**b**) axial magnetic resonance (MR) images. The tumor is located from T7 to T10. (**c**) Coronar and (**d**) sagittal images of selective spinal angiography. The origin of the artery of Adamkiewicz is at level T9 on the left. Surgery was abandoned, and external irradiation was performed.

In three children, surgery was considered too risky, and the treatment method was changed. One patient with local relapse of a hepatocellular carcinoma (Fig. 2) received proton beam therapy, and one patient with neuroblastoma (Fig. 3) underwent conventional radiotherapy. One patient with thoracoabdominal neuroblastoma (patient 9) underwent surgery on the abdominal part of the tumor. At this writing, irradiation of the thoracic part is scheduled. The decision was made by the interdisciplinary tumor board consisting of pediatric radiologists, pediatric oncologists, and pediatric surgeons.

The patient with neurofibroma underwent surgery. It was anticipated that the neurofibroma could be excised with the capsule intact, and thus with a low risk of injury to the AKA. The tumor was successfully removed. No complications occurred during either DSA or surgery.

Tumor recurrence was not observed in the patients who received local therapy (6/10) with a median follow-up period of 25 months (range, 2–94 months) (Table 2). One patient was lost to follow-up evaluation.

**Table 2:** Therapy and follow-up evaluation

Patient	Therapy	Follow-up (months)	Follow-up findings
1	Surgery	0	Lost to follow-up
2	Surgery	33	NED
3	Observation	10	DR
4	Irradiation	94	No local relapse
5	Surgery	46	SD
6	Irradiation	7	NED
7	Surgery	17	SD
8	Surgery	2	SD
9	Irradiation	0.5	N/A
10	Surgery	0.5	N/A

*NED* no evidence of disease; *DR* disease regression; *SD* stable disease; *N/A* not available due to too short follow-up period

## Discussion

This report describes our experience with preoperative selective spinal DSA in surgery for solid tumors of the posterior mediastinum in children and its impact on the therapeutic strategy. In 10 of 36 patients who underwent surgery for posterior mediastinal tumor, the tumor was located left-sided or bilaterally and extended into the vertebral levels of T8 to L1, so spinal DSA was performed. In four patients, the AKA originated at the same level and side as the tumor. To avoid injury to the AKA, the planned surgery was not performed for three of the patients. No complications were associated with surgery or spinal DSA.

Nordin et al.^23^ successfully and safely performed spinal DSA in two children with posterior mediastinal tumors. A systematic review of 11 studies comprising 87 patients receiving spinal DSA for diagnosis, preoperative planning, and therapy in pediatric spinal vascular pathologies reported two post-interventional complications (scrotal swelling and procedure-related subarachnoid hemorrhage).^24^ The data support a positive assessment of spinal DSA for children in general.

Spinal DSA is an invasive method associated with radiation exposure. Therefore, its application should be carefully assessed. We limit it to situations that indicate an increased risk of injury to the AKA, which is extension of the tumor to vertebral levels T8 to L1 and location on the left side.

Vertebral levels T8 to L1 differ from vertebral levels T9 to T12, which are the boundaries in the INRGSS for image-defined risk factors concerning infiltration of the costovertebral junction.^10^ A meta-analysis has shown that 7.3% of the AKAs arise at T8 and 6.9% at L1.^9^ Interestingly, in our cohort, the AKA appeared at L1 in three patients, for whom we thus would not have performed DSA if we had not included this vertebral level.

For diagnostic workup, we applied the diagnostic gold standard of spinal DSA and did not use MRI or CT angiography, which are used for AKA visualization in adults.^25^–^29^ Only 64-section CT is described for children.^30^ However, this method is associated with a potentially higher radiation dose due to the large field of view necessary for diagnosis and the possible misinterpretation of the anterior medullary vein as the AKA in case of non-optimal contrast agent timing. The latter risk exists particularly for children, in whom the vessels generally are smaller than in adults.^30^ Noninvasive methods developed for the demonstration of AKA in adults may be applicable, but to date have not been validated for children.

With modern techniques, spinal angiography can be performed as a very targeted diagnostic procedure, with low radiation exposure focused on the affected vertebral segments and starting on the left side. The relatively high incidence of complications cited by Ou et al.^30^ is based on data reported more than 20 years ago that do not reflect the current clinical and technical standard. Due to the precise localization of the origin of AKA in relation to the tumor, spinal DSA remains the diagnostic method of choice.

## Conclusions

Spinal DSA can be safely and reliably applied for preoperative AKA demonstration in children with a posterior mediastinal tumor. It allows a valid assessment of the surgical risk and thus may prevent an injury to the AKA. Performing DSA for left-sided tumors extending to vertebral levels T8 to L1, the most common region of AKA location, avoids its unnecessary use.
